# Epilepsy

**Published:** 1864-04

**Authors:** William M. Cornell


### CORRESPONDENCE.

### EPILEPSY. BY WILLIAM M. CORNELL, M. D. L L. D.

To the Editor of the *Buffalo Medical and Surgical Journal:*

DEAR SIR:—I have been much interested in reading an article in the December number, 1863, and also a continuance of the same in the January number, 1864, of your journal, upon Epilepsy, translated from the German of Dr. Finkelnburg, of the University of Bonn, Germany, by H. Lassing, M. D., of New York. Dr. Lassing has done good service to the profession by this translation, and quite as much by his own notes. He deserves the thanks of us all.

For twenty years I have been studying the pathology and the best manner of treating epilepsy, epileptiform, and other analagous diseases, which are usually called *nervous.* I have for the last five years confined my practice almost exclusively to these cases. I have written my views of the pathology and treatment of this whole class of diseases very fully in the medical periodical journals of the day. The cases treated by *digitalis,* referred to by Dr. Lassing, in his notes, as published in the Charleston *Medical Journal,* vol. XVIII,, May, 1858, is but one of these articles published in that journal. There are several others there. But the more thorough discussion of this subject may be found in vol. LI. of the Boston *Medical Journal*, 1855. There your readers will find, under the caption of "Observations on Epilepsy," pages 75–159–220–241, 256–316–373 and 342, my views very fully expressed. These were afterwards published in a pamphlet, which is now out of print. During the last five years, since I have resided in Philadelphia, I have written, in the form of correspondence, for the *Medical and Surgical Reporter*, of Philadelphia, a number of articles upon the "Cause of Epilepsy," which, I presume, you have read.

More than five hundred cases of epilepsy and epileptiform diseases have come under my treatment, since I had some reputation in this disease. I say *disease*, though I do not believe epilepsy to be a disease, strictly speaking, but rather the *convulsive manifestation* of other diseases, wounds, etc.

Just previous to the outbreaking of the present rebellion, a clergyman, of the Baptist denomination, came to me from Charleston, S. C., to be treated. You will oblige me by publishing, in this connection, a letter which he wrote of his own accord, and published in their denominational paper of this city, the *Baptist Chronicle*. It is as follows:

The undersigned, a native of Charleston, S. C., had been an epileptic for several years, and his attacks were very severe, exhausting the skill of the ablest physicians of the country, and as eminent, perhaps, as any country can produce, without relief. About a year ago, his attention was called to two articles which appeared in the Charleston *Medical Journal*, from the pen of Dr. W. Cornell, formerly of Boston, and now of Philadelphia, on the subject of Epilepsy. He forthwith opened a correspondence with Dr. C., and received such assurance from him and others as led him to visit Philadelphia, and place himself under Dr. Cornell's treatment. He began to improve immediately, and he believes his improvement has been radical and permanent. He has not had an attack since, now nearly one year, nor anything approaching one, except a little vertigo once or twice, which passed off in a few minutes without any unpleasant effect. He has been a minister of the Gospel all his life and has been actively engaged in the labors thereof, until he became an epileptic: since then he has been compelled to withdraw therefrom. He fondly hopes now, being so much improved, he shall be able to resume his loved work at no distant day. We may be permitted to remark here that Dr. Cornell is a regular bred physician of the Old School, and a Christian gentlemen of high standing, and may be implicitly relied upon. He (Dr. C.) has treated, perhaps, in the course of a long practice, not less than five hundred epileptics, drawn to him from every portion of this widely extended country. All of these have not been cured. This was not to be expected. There are, undoubtedly, cases that are beyond the reach of human skill, but many of these have been more or less improved, and the majority of all have been radically and permanently cured, *i. e.* they have never had a fit since, and many of them after the lapse of many years. The following case is given, selected from many other similar cases that might be quoted, in proof of this statement:

"I feel constrained by a sense of gratitude, and also a desire to benefit others who may be similarly afflicted, to acknowledge, through your columns, the relief I have gained by the use of medicine prepared by Dr. William Cornell, of Boston. For about seventeen years I have been subject to violent attacks of convulsion. They occurred at intervals, varying from two to seven weeks—the fits succeeding each other sometimes to the number of seven or eight. During that time I had been under the treatment of several eminent physicians of Boston and vicinity, sometimes following the directions of one for a year without relief. I have applied to the McLean Hospital, tested the efficiency of Thomsonianism for thirteen weeks the Homœopathic system for two months; and so desirable was health, that I even resorted to Mesmerism to disclose the cause of the difficulty and prescribe the remedy, but still my fits continued.

"Hearing of Dr. Cornell's success in similar cases, I called on him in September last, since which time I have taken his medicine and carefully followed his directions with the exception of a single occasion. On the 5th of January last, being absent from home, I neglected to take medicine, and owing to that circumstance, together with some degree of excitement, I had a slight attack. From that time I have enjoyed unusual health, and have since had no symptoms of the complaint that has probably caused me more suffering than would be experienced in a hundred deaths.

"N. B.—Any information will be gladly given by the subscriber.

"WILLIAM T. PAGE.

"*East Stoughton, Mass., April,* 1849."

The writer has seen a letter from Mr. Page, the party referred to, written by him within ten days past, reaffirming that up to this moment he enjoys the most perfect health, never having another fit.

Dr. Cornell resides at No. 1722 South Penn Square, Philadelphia, where he may be consulted by victims of this terrible disease who may desire relief, and the aid of his professional skill. C. M. BREAKER.

*Philadelphia, April* 10, 1860. —*Bap. Chron.*

These are *bona fide* letters, and were I in full general practice I would not allow them to be published; but, as I am not, I see no more objection to their being published than there would be to publishing the success of a Medical College as respects the number of its pupils. You, of course, are at liberty to do as you please with them; as you, also, may with the articles referred to in the journals just named. They are before the profession, and they will judge of them as they please. I have never withheld a full and free disclosure of all the treatment, and of all the medicines used, with the manner of compounding them, and have always consulted freely with all members of a State society and of the American Medical Association, of which I am a permanent member. The main idea, as diverse from that of the faculty generally, when the articles for the "Boston Medical Journal" were written was, that the *primary* or *original cause* of Epilepsy *was in the blood;* some abnormal state or poisonous ingredient therein; and, while this was the *original cause*, the immediate exciting causes were manifold; such as *worms, excessive venery, gluttony, fright, &c., &c.*

When that idea was first published, I was not aware that a single Physician agreed with me in such an opinion, and you may be assured when some five years after it was published, I found myself summoned to Salem, Massachusetts, with Dr. Bell, (then late of the McLean Hospital for the Insane at Charlestown, and at that time President of the Massachusetts Medical Society,) *as experts*, in a case there pending between a man who had sued the city for damages, because his horse had fallen through a bridge, thrown him from his chaise and shaken him, so that ever after he had epeliptic attacks. We were summoned by the complainant. The attorney for the city was one of your slow-healing, saucy fellows, who manifested a disposition to castigate Dr. Bell upon the witness-stand, but in every instance he was completely floored by the Doctor, often to the great amusement of the spectators and of the Court itself. Is epelipsy an organic disease? said the attorney. The doctor, naturally tall, straightening himself up to his full height, replied, "Well, sir, that depends upon what you call the *blood*—if *that* is an organ, then I would say epilepsy *is* an organic disease, for I consider it a disease of the blood."

Pardon me for referring to this trial, for it was just the reverse of most cases, where medical men are made the mere sport of the legal profession. The lawyer was really the sport of the Doctor. To one question which the lawyer asked, Dr. Bell replied, "It would not be possible for me to answer or explain this matter, that an *unprofessional* man could comprehend it." This was the last question, and the impudent attorney felt that for once, he had been rendered *hors du combat.* As said, I was gratified to find my opinion agreed in by so eminent a man; and among the worthies who have fallen victims in this accursed rebellion may be classed, in the foremost ranks, the same Dr. Bell.

Now, sir, I have said all that I wish to say in this paper. If you wish it, I may write more hereafter, and I propose as soon as I can find time to do so, to embody all that I have written, with some things that I have not yet written, in a book for the reading, (I will not say for the improvement) of the Profession, for I do not feel competent to instruct them. But I do hail with pleasure anything upon the subject of Epilepsy, as I did your report from Dr. Lassing.

